# The importance of a medical chaperone: a quality improvement study exploring the use of a note stamp in a tertiary breast surgery unit

**DOI:** 10.1136/bmjopen-2014-007319

**Published:** 2015-07-15

**Authors:** K Rose, S Eshelby, P Thiruchelvam, A Khoo, K Hogben

**Affiliations:** 1 West Middlesex University Hospital NHS Trust, London, UK; 2 Charing Cross Hospital, Imperial College Healthcare NHS Trust, London, UK; 3 The Royal Marsden NHS Foundation Trust, London, UK; 4 Norfolk and Norwich University Hospital, NHS Foundation Trust, London, UK

**Keywords:** Chaperone, Intimate examination, Breast Examination, Quality Improvement

## Abstract

**Objectives:**

The project aim was to determine current use and documentation of medical chaperones within a major breast service unit. It explored ways of improving adherence to professional guidelines concerning chaperones.

**Setting:**

The single centre quality improvement project was completed in a tertiary breast service unit in North West London. It was a three-stage project with initial audit in October 2013, 1st postintervention cycle in November 2013 and 2nd postintervention cycle in October 2014.

**Participants:**

In each study cycle, data were collected from entries in clinic notes until at least 155 encounters with documented clinical examination were analysed. All notes were of female patients.

**Interventions:**

(1) Intervention 1st cycle: presentation and discussion of chaperone guidelines alongside reminder posters and introduction of note stamp. (2) Intervention 2nd cycle: note stamp alone.

**Primary and secondary outcome measures:**

Documentation of chaperone offer, documentation of patient preference regarding chaperone, identifier (name or signature) of chaperone present and gender of examining clinician.

**Results:**

In the 1st postintervention cycle, 69.95% documentation of chaperone offer was recorded, p<0.001, CI (59.04% to 80.76%). This result was replicated in the 2nd postintervention cycle a year later with 74.86% documentation of chaperone offer recorded, p<0.001, CI (66.41% to 83.31%). The 4.91% difference was insignificant; p=0.294, CI (14.03% to 4.21%).

**Conclusions:**

The authors suggest that a proforma approach to medical chaperones is an effective means of ensuring adherence to best practice guidelines. A stamp, or similar, that can be embedded into documentation structure is an effective example of such an approach. Improved documentation allows any problems with adherence to guidelines to be more easily identified, helping to ensure the safeguarding of patients and staff involved in intimate examinations.

**Strengths and limitations of this study**

- This quality improvement study allowed us to comment on the efficacy of our note stamp intervention as an isolated tool as well as part of an ‘intervention package’ in improving offering and documentation of chaperone. Conclusions could thus be drawn on the independent efficacy of the note stamp tool alongside demonstrating reproducibility of results aiding a cause-effect claim.
- Clear professional guidelines were available to provide set standards for defined outcome. The data were collected and analysed by individuals not involved in the process being investigated, which helped ensure validity of results.
- A limitation in this design was the inability to comment on use of chaperone independently to offer and documentation of use. The authors cannot comment on whether this tool increases overall use or if the change is purely in improving documentation.
- In addition, this looked only at chaperone use in the context of breast examination in a single centre in which all patients were female. It is therefore difficult to support an author assumption that these results are applicable across all intimate examinations; further work in different centres and examination type would be valuable.

## Background

A medical chaperone is an impartial observer present during a consultation between a doctor or allied health professional and a patient. A medical chaperone acts as an advocate for the patient and can help patients understand exactly what is happening, and why. Increasingly, chaperones are seen as important from a medicolegal perspective, as protection for the clinician against unjust allegations but also being prepared to raise concerns about a clinician's behaviour and action if they deem them to be inappropriate.

In the UK, medical practitioners are provided with clear professional guidance on appropriate use of chaperones. The General Medical Council (GMC), the Royal College of Nursing, the NHS Clinical Governance Support Teams and the medical defence organisations have all generated guidance around chaperoning.^1–4^ In the 2013 update of Good Medical Practice, Intimate Examinations and Chaperones formed a key part of the Maintaining Boundaries section. These guidelines set out the role of a chaperone as patient advocate as well as doctor protection; it clarifies that chaperones should be considered if it is necessary to perform any kind of intimate examination; “This is likely to include examinations of breasts, genitalia and rectum, but could also include any examination where it is necessary to touch or even be close to the patient.”^1^

Recent studies have indicated insufficient use of chaperones across primary as well as secondary care.^5–8^ Previous studies have highlighted how doctors’ attitudes to intimate examination may influence their use of chaperones.^9^ ^10^ Studies have also shown that many patients see the offer of a chaperone as a sign of respect from their doctor and helps build a good patient–doctor relationship.^5^ ^6^

In this project, the authors aimed to investigate current offer, documentation and, thus, use of chaperones, within a major breast service unit. It then explored the use of a ‘note stamp’ to improve adherence to professional guidelines concerning chaperones.

## Methods

**Figure 1:**
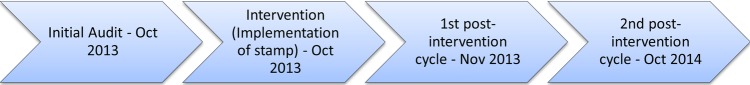
Study timeline.

This single centre quality improvement project included three audit cycles (figure 1). All patients attending a breast service department outpatient appointment were included. It is important to note that this does not include patients attending routine breast screening.

Sample size was calculated based on an estimated average number of clinical encounters per week within the department; population number 250. At 95% confidence levels and an interval of ±5, the sample size needed was 152. This was achieved across all cycles.

An initial retrospective analysis of all available outpatient notes after clinical completion was performed. In all three cycles, patient gender was recorded but further demographic analysis was not. The authors audited four initial questions to determine full adherence to professional guidelines. Full guideline adherence is defined as documentation of chaperone being offered, documentation of patient preference for chaperone and chaperone identifier (name or signature of chaperone) present.^1^

1. Was an examination documented?
2. Was offer of chaperone documented in the notes?
3. Was documentation of chaperone preference indicated in the notes—declined/accepted?
4. Was the attending doctor male or female?

### First postintervention cycle

The following interventions were completed and the department was re-audited.

*Interventions*

**Figure 2:**
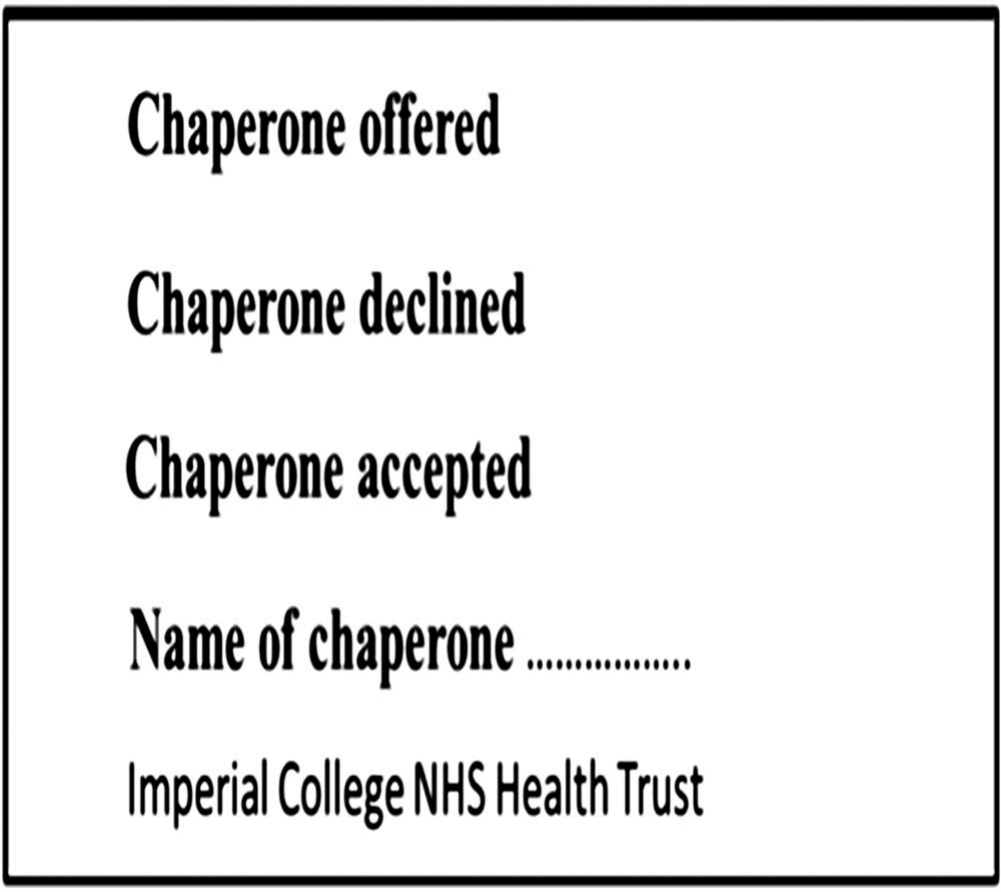
Initial Chaperone Stamp.

- Discussion of findings in the weekly multi-disciplinary team (MDT) meeting and recent GMC guidelines regarding “intimate exams and chaperone” were highlighted. Additionally, data from a freedom of information request to the Medical Protection Society (MPS) quoted were “at least twelve cases in 2012, where criminal investigations were instigated against doctors around sexual assault.” (Godeseth C, personal communication October 2013).
- Introduction of a ‘chaperone stamp’ into the outpatient notes (figure 2).
- Memo pamphlets placed in clinic rooms (see online supplementary appendix 1).

The same questions plus an additional two questions were then re-audited.

1. Was an examination documented?
2. Was the note stamp present?
3. Was offer of chaperone documented in the notes?
4. Was documentation of chaperone preference indicated in the notes—declined/accepted?
5. If documented as present, was a name/signature present?
6. Was the attending doctor male or female?

### Second postintervention cycle

**Figure 3:**
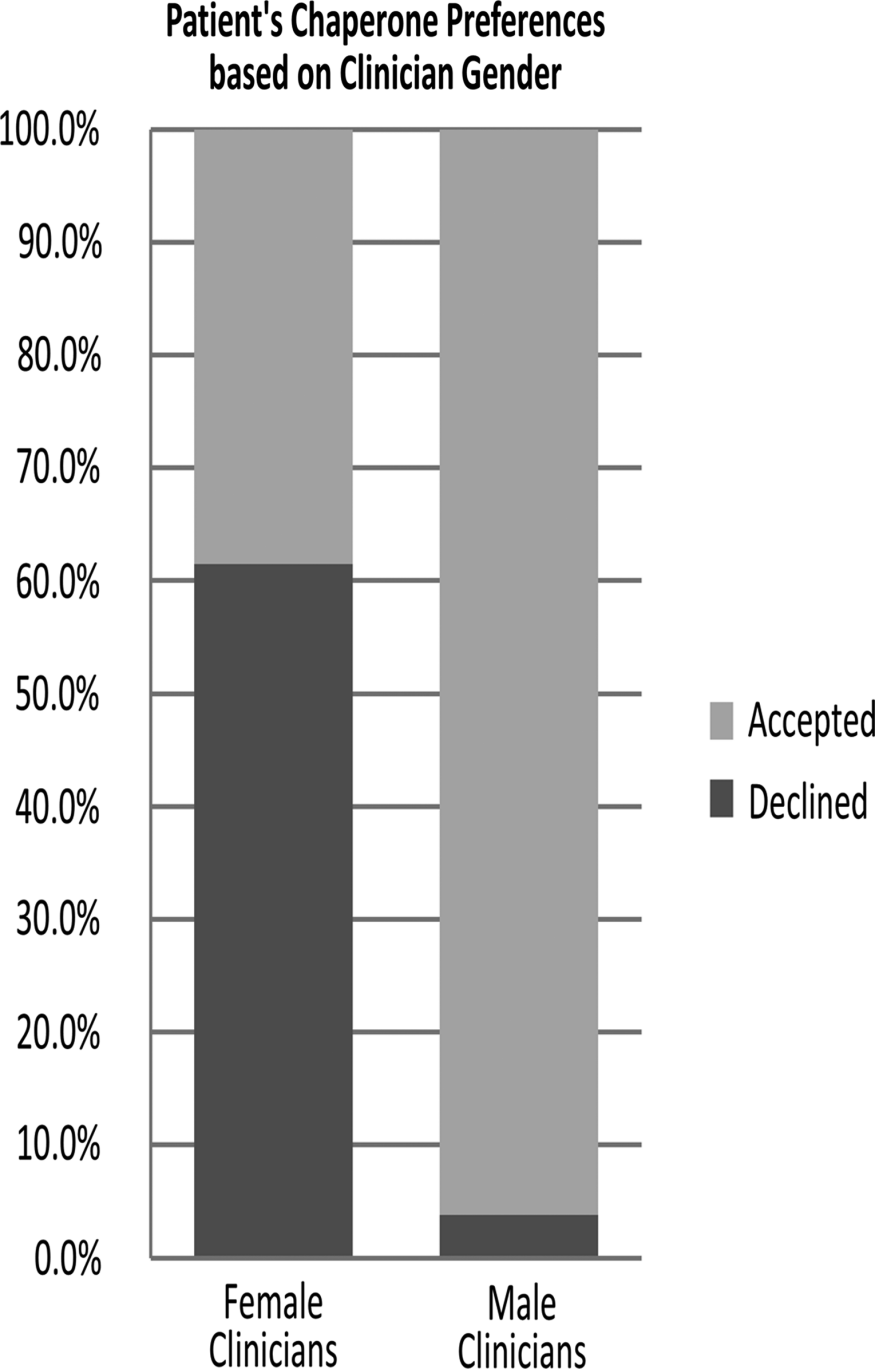
Graph demonstrating patient preference for chaperone compared to clinician gender.

A second postintervention cycle was completed a year after the initial audit. This cycle focused solely on the addition of the ‘chaperone stamp’. No further discussion or re-education about chaperone importance was performed at this time. The same questions were audited (figure 3).

Data were collected and inputted into Microsoft Excel and analysed using a two-tailed z-test using the open access statistics calculator available through http://www.socscistatistics.com/tests/ztest/Default2.aspx.

## Results

Initial audit demonstrated failure of chaperone documentation in all cases. Consultations without clinical examination/no record of consult in notes (n=22). These were excluded from further analysis. Consultations in which a chaperone should have been offered and preference documented (n=161). Consultations where this occurred (n=0).

### Main findings

In the first postintervention cycle, 69.95%, p<0.01, CI (59.04% to 80.76%) documentation of chaperone offer was present. This result was replicated a year later with 74.86%, p<0.01, CI (66.41% to 83.31%) documentation of chaperone offer present. The 4.91% difference was insignificant, p=0.294, CI (14.03% to 4.21%).

In the second postintervention cycle a year later, stamp use was not explained to clinicians. This resulted in a significant decrease in valid chaperone identifiers for cases where chaperone was accepted. In the first postintervention cycle 98.21%, p<0.01, CI (94.98% to 100%) of cases when chaperones were documented as accepted also had a valid identifier. In the second postintervention cycle this stood at 40.63%, p<0.01, CI (27.72% to 53.54%); a significant 57.88% fewer, p<0.01, CI (47.75% to 68.01%). Complete guideline adherence in total, including cases when chaperone was declined, stood at 56.59%.

It is more likely for a female patient to decline a chaperone if the clinician is also female; 61.45%, p<0.01, CI (47.69% to 75.21%) of women who were offered a chaperone by a female clinician declined compared to 3.82%, p<0.01, CI (0.17% to 7.47%) with male clinicians; 57.63% difference between sexes, p<0.001, CI (46.80% to 68.46%) (figure 2).

### Additional findings

#### First postintervention cycle

Chaperone name or signature was present in 108 of 112 cases where chaperones were documented as accepted. Two patients accepted chaperones but were then documented as not available; these two patients were then happy to go ahead without chaperones and have been included as having a valid identifier.

In cases in which an examination was documented but the stamp was not present (n=22), a clinician had documented chaperone use by hand in two cases.

#### Second postintervention cycle

Chaperone name or signature was correctly documented in 39 of 96 cases, in 26 cases lead clinician signature was present and the remaining 31 were left blank. Two patients accepted chaperones but were then documented as not available; these two patients were then happy to go ahead without chaperones.

In cases where an examination was documented but the stamp was not present (n=6), a clinician had documented use by hand in one case.

## Discussion

This project highlighted important points around the offer and documentation of use of chaperones during breast examination. This study presented an inferior picture to that found by Allberry and Fernando^7^ in 2012; 24% of intimate examinations in a sexual health clinic had documentation of chaperone preference. The authors highlight the phrase “if it's not documented it didn't happen,” 0% documentation rate was considered a concerning record of practice.

Availability of a chaperone has previously been cited as a key influence in a clinician's decision to offer a patient this facility.^5^ The authors speculate that clinicians may feel it better not to offer a chaperone than to offer and have to explain that, unfortunately, no one is available. Further research in this area would be beneficial. This study did not seek to try and address the constraints of ‘chaperone availability’, although would like to report that those in the department involved were surprised to find how often a chaperone was available when required. The authors would suggest that a chaperone stamp tool, and/or other means of encouraging good documentation habits, will provide departments with clear data. In an increasingly paperless National Health Science, an electronic equivalent could be introduced into outpatient templates; a pop up box, once documentation of intimate examination is entered, may be effective. This in turn will allow a better understanding of where resources are really lacking and ensure efficient re-distribution if necessary. This will also help to ensure that where a chaperone is not available, patients are informed and are given the option to decline or to continue examination, after discussion with their clinician.

The authors argue that the culture of chaperone use needs to change; the focus should be on each individual patient decision and not on clinician presumption of preference. Although interesting work exploring why clinicians do/do not offer chaperones has been completed,^8^ ^10^ we would suggest that clinician preference is no longer of relevance. In the UK, clear professional guidance has been issued by the GMC^1^: patient preference can only be ascertained by offering a chaperone and thus not doing so is a difficult judgement to defend. This information will be important to female clinicians who have traditionally less frequently offered a chaperone during breast examinations.^6^ ^8^ ^11^ ^12^ This study, as others,^12^^–^14 demonstrates that women are more likely to decline the offer of a chaperone when the examining clinician is female; 61.45% declined a chaperone offered by a female clinician compared to 3.82% declining if the clinician was male. The reverse of this is that nearly 40% of women will still choose a chaperone even with a female clinician.

Areas where the authors felt that the intervention of a ‘chaperone stamp’ could make the biggest difference were in reminding clinicians to offer a chaperone and in ensuring and expediting documentation of chaperonage.

The conceptualised chaperone stamp was a simple, easy and cheap intervention to implement and required very little extra input from non-clinical staff, which was viewed as advantageous. It was also unobtrusive, in the hope that clinicians were reminded of their obligation to offer and document a chaperone without feeling forced to do so. The process was also time effective as minimal extra notation was required. It is not possible to comment on whether clinicians were offering but not documenting chaperone use before this study; comment on whether this intervention changed practice in offering or only in documenting is not possible. It was, however, clearly effective in ensuring adequate documentation of chaperone offer, patient preference and chaperone identifier.

The results of the first postintervention cycle demonstrated that the combination of discussion, re-education, pamphlet reminders and a note stamp, significantly improved the correct use of chaperones in this department. The use of chaperones after these interventions compared very favourably to all other literature looking at breast and other intimate examinations.^5–9^ ^11^ The per cent offer rate across both genders of clinician is the highest found within the literature to date.

In a health environment where changes are frequently made on too little evidence, a decision for a second postintervention cycle was made. This demonstrated a similar rate of chaperone offer and documentation.

However, of important note, after the second postintervention cycle, we had hoped to be able to demonstrate that the stamp alone was enough to ensure full compliance with GMC guidelines. The authors would argue that it has proved its value in ensuring chaperones are offered to patients and that this is documented. However, it became clear that there was some confusion around who should sign the stamp, with 26 of the 96 offers having the lead clinician's signature rather than that of the chaperone. The authors are in discussion with the participating trust to ensure this becomes a permanent addition to patient notes within this department before thinking about wider-reaching applications across a range of outpatient settings. Ensuring chaperones can be identified will need to be highlighted (as in the first postintervention cycle) by any departments keen to start using this tool.

Newer research has started to look at why different clinicians have different practice around the use of chaperones.^8^ ^10^ Given the clarity of current clinical guidance,^1–4^ the authors suggest that discussion in this area should not focus on whether or not chaperones are used but, instead, focus should be on how to ensure chaperone offer and documentation become routine practice. Many departments,^11^ including the one in this study, have clear chaperone policies. The authors would suggest that the key factor is to ensure that clinicians are empowered to easily follow policy and practice. In discussion during this study and echoed by Price *et al*^5^, availability of chaperones, in addition to remembering to offer, was the other key barrier to consistent use. Although this study did not encompass resourcing issues in providing extra staff for chaperoning, the introduction of a tool to encourage the culture of effective documentation will allow departments to more efficiently plan for gaps in services and redeploy current resources more efficiently. It also ensures that when chaperones are not offered, this is easily identified and can be investigated and addressed (table 1).

**Table 1:** Summary of three cycles of audit and the uptake of medical chaperones

	Clinician gender	Initial audit	First postintervention cycle	Second postintervention cycle
Total consultations		183	214	202
Total documented examinations		161	193	175
	M	123	124 (64.25%)	126 (72.00%)
	F	38	69 (35.75%)	49 (28.00%)
Chaperone offer documented		0	135 (69.95%)	131 (74.86%)
	M	0	93 (75.00%)	90 (71.43%)
	F	0	42 (60.09%)	41 (83.67%)
Chaperone accepted (of those offered)		NA	112 (82.96%)	96 (70.99%)
	M	NA	90 (96.77%)	86 (95.56%)
	F	NA	22 (52.38%)	10 (24.39%)
Chaperone identifier present		NA	110 (98.21%)	39 (40.63%)
Chaperone declined (of those offered)		NA	23 (17.04%)	35 (26.32%)
	M	NA	3 (03.23%)	4 (4.30%)
	F	NA	20 (47.62%)	31 (75.61%)
Full guideline adherence observed		0	133 (68.91%)	74 (56.49%)

F, female; M, male; NA, not applicable.

The authors would suggest that a proforma approach to clinical note structure, whether a stamp, sticker or electronic form section, is an effective way to encourage the culture of chaperonage as well as providing better data to ensure services can be restructured if necessary. It is cautioned that this study cannot comment on the longevity of the effect of such an intervention and further work would need to be carried out to prove lasting efficacy. Additionally work across other centres and looking at whether the tool remains effective with a broader patient group, particularly including male patients, would also be helpful. Until such work is completed, a stamp forms a particularly cheap and easy tool for any department to use initially.

Interesting work is being carried out to help understand why clinicians do/do not offer chaperones.^8^ ^10^ It could be cautioned that this approach may result in a culture shift that is too slow, considering the clarity of the current guidelines. A change such as this allows a 75% compliance rate; exploration of why some consultations lack offer of chaperone can then be carried out in the remaining 25% of cases.

## Conclusion

In a modern healthcare environment, where patient choice and autonomy are paramount, it is essential that clinicians and departments regularly performing intimate examinations fully comply with chaperone guidance. The authors would suggest that a proforma approach that can be embedded into documentation structure, such as our chaperone stamp, is an effective way to ensure compliance with guidelines. Improved documentation allows any problems with adherence to guidelines to be more easily identified, helping to ensure the safeguarding of patients and staff involved in intimate examinations.

## References

[1] General Medical Council (GMC). Intimate examinations and chaperones—Code: GMC/IEC/0313. March 2013.

[2] Medical Protection Society (MPS). Factsheet Chaperones 2014 http://www.medicalprotection.org/mps-england-chaperones-factsheet.pdf accessed July 2014

[3] Medical Defence Union (MDU). Someone to watch over me. MDU J 2014;30:11–13.

[4] Royal College of Nursing (RCN). Chaperoning: the role of the nurse and the rights of patients Guidance for nursing staff 2006 https://www.rcn.org.uk/__data/assets/pdf_file/0006/78513/001446.pdf—Publication code 001 446, republished June 2006 (accessed Jul 2014).

[5] Price DH, Tracy CS, Upshur RE Chaperone use during intimate examinations in primary care: postal survey of family physicians. BMC Fam Pract 2005;6:52 10.1186/1471-2296-6-52 16371153 PMC1360073

[6] Sinha S, De A, Jones N Patients’ attitude towards the use of a chaperone in breast examination. Ann R Coll Surg Engl 2009;91:46–9. 10.1308/003588409X358971 PMC2752243 18990268

[7] Allberry C, Fernando I An audit of chaperone use for intimate examinations in an integrated sexual health clinic. Int J STD AIDS 2012;23:593–4. 10.1258/ijsa.2012.012018 22930299

[8] Jones K, Biezen R, Beovich B Chaperones for intimate examinations in family medicine: findings from a pilot study in Melbourne, Australia. Med Sci Law 2015;55:6–10. 10.1177/0025802413518318 24477199

[9] Guidozzi Y, Gardner J, Dhai A Professionalism in the intimate examination: how healthcare practitioners feel about having chaperones present during an intimate consultation and examination. S Afr Med J 2012;103:25–7. 23237119 10.7196/samj.6224

[10] Hine P, Smith H Attitudes of UK doctors to intimate examinations. Cult Health Sex 2014;16:944–59. 10.1080/13691058.2014.923584 24992376

[11] Loizides S, Kallis A, Oswal A Chaperone policy in accident and emergency departments: a national survey. J Eval Clin Pract 2010;16:107–10. 10.1111/j.1365-2753.2009.01122.x 20367821

[12] Nkwo PO, Chigbu CO, Nweze S Presence of chaperones during pelvic examinations in southeast Nigeria: women's opinions, attitude, and preferences. Niger J Clin Pract 2013;16:458–61. 10.4103/1119-3077.116889 23974739

[13] Conway S, Harvey I Use and offering of chaperones by general practitioners: postal survey in Norfolk. BMJ 2005;330:235 10.1136/bmj.38320.472986.8F 15604154 PMC546073

[14] Rosenthal J, Rymer J, Roger Jones W Chaperones for intimate examinations: cross sectional survey of attitudes and practices of general practitioners. BMJ 2005;330:234 10.1136/bmj.38315.646053.F7 15579477 PMC546072

